# Molecular Mechanisms of Phytochemicals from Chaga Mushroom (*Inonotus obliquus*) Against Colorectal Cancer: Insights from Network Pharmacology, Molecular Docking, and Bioinformatics

**DOI:** 10.3390/ijms26167664

**Published:** 2025-08-08

**Authors:** Yingzi Wu, Jiayin Liu, Jinhai Luo, Baojun Xu

**Affiliations:** 1 Food Science and Technology Program, Department of Life Sciences, Beijing Normal-Hong Kong Baptist University, Zhuhai 519087, China; wuyingzi@uic.edu.cn (Y.W.); liujiayin0923@163.com (J.L.); luojinhai@uic.edu.cn (J.L.); 2 School of Chinese Medicine, Hong Kong Baptist University, Hong Kong, China

**Keywords:** Chaga mushroom, *Inonotus obliquus*, bioactive compounds, colorectal cancer, mechanisms, network pharmacology, bioinformatics

## Abstract

This study aimed to explore the molecular mechanisms of phytochemicals from Chaga mushroom (*Inonotus obliquus*) against colorectal cancer (CRC) using a combination of network pharmacology, molecular docking, and bioinformatics. Active components and targets of Chaga mushroom and CRC were collected from databases. A drug-compound-target-disease network was constructed, and protein–protein interaction (PPI) analysis was performed to identify core targets. KEGG and GO enrichment analyses were conducted to elucidate the involved pathways. Molecular docking estimated the binding affinities of key compounds to their targets, and bioinformatics analysis assessed differential gene expression and immune infiltration. The study identified 26 bioactive compounds and 244 potential targets. Core targets included AKT1, IFNG, and MMP9. Molecular docking showed strong binding affinities, and bioinformatics analysis revealed significant differential expression and immune infiltration patterns. These findings suggest that Chaga mushroom phytochemicals may exert anticancer effects through multiple pathways, highlighting their potential as novel CRC treatments. This study provides a comprehensive understanding of the molecular mechanisms underlying the anticancer effects of Chaga mushroom phytochemicals on CRC. Future research should focus on experimental validation and further exploration of their therapeutic potential.

## 1. Introduction

Colorectal cancer (CRC) is a leading cause of cancer-related mortality worldwide, posing significant challenges to public health [1]. Despite advances in surgical techniques, chemotherapy, and targeted therapies, the prognosis for CRC patients remains poor, with high recurrence rates and limited treatment options for advanced stages [2]. Therefore, there is an urgent need to explore novel therapeutic strategies and agents to improve the outcomes for CRC patients.

In recent years, natural products have garnered increasing attention as potential sources of anticancer agents [3]. Mushrooms have a long history of promoting health in Asian countries, especially in China and Japan [4]. Due to the relatively easy administration of some mushroom preparations, they are more easily accepted by people. Among them, Chaga mushroom (*Inonotus obliquus*) has shown promising health benefits, including antioxidant, anti-inflammatory, and immune-regulating properties [5]. These properties have been extensively studied, and Chaga mushroom has been used in traditional treatments in Eastern Europe and Asia due to its various pharmacological effects [6]. Therefore, many cancer patients even use Chaga mushrooms as a supplementary medication during chemotherapy or radiation therapy [7]. However, few studies have delved into the molecular mechanisms and therapeutic targets of Chaga mushrooms in the treatment of colorectal cancer. This gap in knowledge highlights the need for comprehensive research to explore the potential anticancer effects of Chaga mushroom phytochemicals on CRC.

Network pharmacology, molecular docking, and bioinformatics have emerged as powerful tools for exploring potential mechanisms of disease and bioactive compounds of medicine [8]. Network pharmacology allows for the systematic analysis of the interactions between multiple phytochemicals and their potential targets within the complex biological network of cancer cells [9]. Molecular docking can predict the binding affinity and interaction modes between specific phytochemicals and their target proteins at the atomic level [10]. Bioinformatics techniques enable the comprehensive analysis of gene expression data, protein–protein interaction, and signaling pathways to uncover the key molecular events involved in the anticancer effects [11]. By combining these multidisciplinary approaches, researchers can effectively narrow down the scope of potential targets and mechanisms, predict potential effective targets, pathways, and molecular mechanisms for drug therapy of diseases, simulate the binding affinity of drugs with specific proteins, and explore the impact of specific genes on disease prognosis [12].

In this study, we aim to elucidate the molecular mechanisms of phytochemicals from Chaga mushroom against colorectal cancer, leveraging the insights gained from network pharmacology, molecular docking, and bioinformatics studies. By integrating these multidisciplinary approaches, we hope to explore the potential phytochemicals of Chaga mushroom and identify the related therapeutic targets for the development of CRC treatments. This study will provide strong theoretical support for the mechanism exploration of the anti-colorectal cancer effect of Chaga mushroom bioactive compounds.

## 2. Results

### 2.1. The Bioactive Compounds of Chaga Mushroom and Their Target Proteins

**Figure 1:**
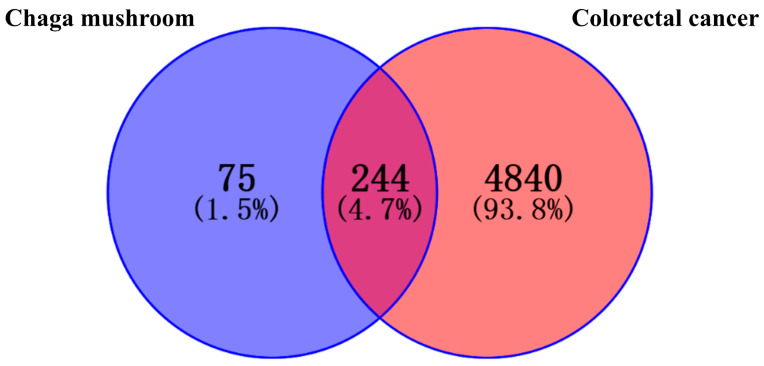
The Venn diagram of intersection targets of Chaga mushroom bioactive compounds and colorectal cancer. The overlap colors indicate the related targets of Chaga anti-colorectal cancer.

Through the literature, 26 active compounds were finally obtained from Chaga mushroom including quercetin, epigallocatechin-3-gallate, kaempferol, myricetin, isorhamnetin, salicylic acid, tangeretin, betulinic acid, oleanolic acid, ellagic acid, 3,4,5-trihydroxybenzoic acid, protocatechuic aldehyde, ferulic acid, catechin, protocatechuic acid, p-coumaric acid, ergosterol, vanillic acid, caffeate, p-MCA, lanosterol, ergosterol peroxide, trametenolic acid, epicatechin gallate, and cedar acid (Table 1). Moreover, a total of 319 targets for Chaga mushroom were collected from the SWISStargetprediction database based on the bioactive compounds identified from the literature. The 5064 colorectal cancer-related targets were obtained from GeneCards, OMIM, and DrugBank databases using the keywords “colorectal cancer” and “colon cancer.” These two kinds of targets were interacted by the Venne diagram (Figure 1), which are the anti-colorectal cancer targets of the Chaga mushroom.

### 2.2. Construction of the “Drug Active Ingredient Target” Interaction Network of Chaga Mushroom

**Figure 2:**
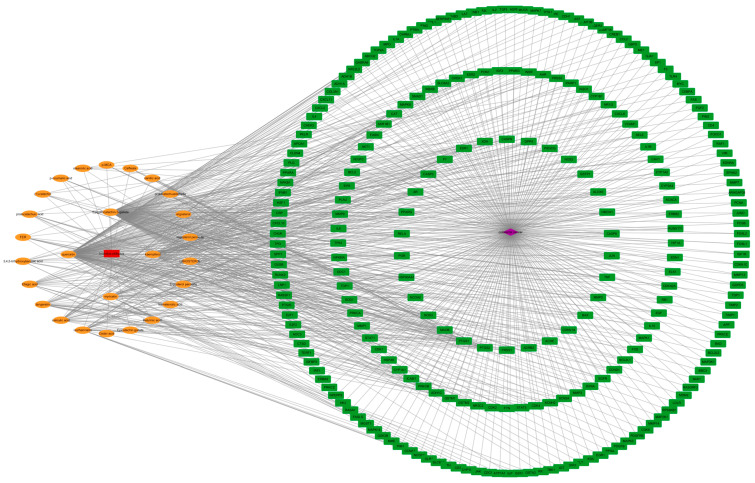
Drug-compound-target-disease network diagram of Chaga mushroom and colorectal cancer. The red square represents Chaga, the orange ovals represent the active compounds of Chaga, the purple spirituality represents colorectal cancer, and the green rectangles represent the targets of Chaga in treating colorectal cancer.

The drug-compound-target-disease network diagram was constructed to visualize the interactions between the active compounds of Chaga mushroom and their potential targets in colorectal cancer (Figure 2). This network comprises 272 nodes and 1362 edges, where nodes represent either compounds or targets, and edges represent the interactions between them. Specifically, the network includes 26 nodes representing the bioactive compounds identified from Chaga mushroom and 244 nodes representing the potential targets. The edges connecting these nodes indicate the predicted interactions between the compounds and their targets.

**Figure 3:**
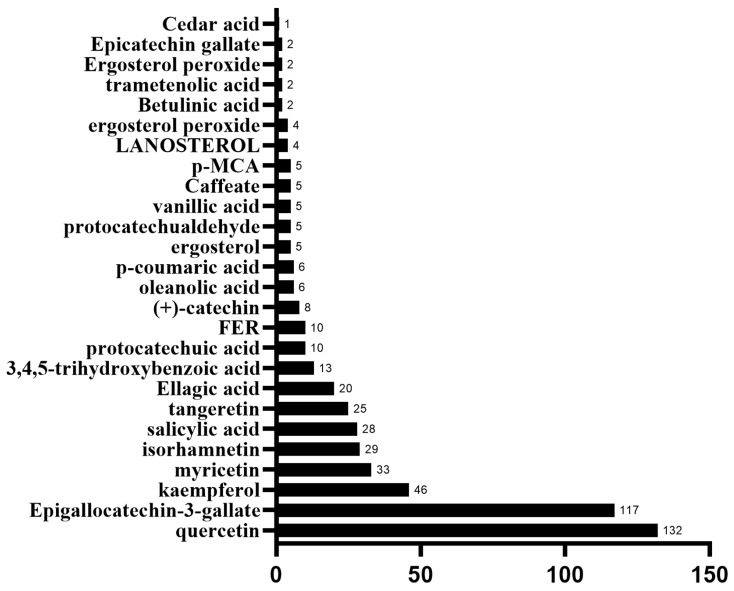
Bioactive compounds from Chaga mushroom ranked by degree.

The degree centrality (DC) values of the 26 compounds were calculated to assess their connectivity within the network (Figure 3). The top five compounds with the highest DC values are quercetin, epigallocatechin-3-gallate, kaempferol, myricetin, and isorhamnetin. These compounds have the highest number of connections to target nodes, suggesting that they may play a significant role in the anticancer effects of Chaga mushroom.

### 2.3. The Protein–Protein Interaction (PPI) Analysis and Target Screening

**Figure 4:**
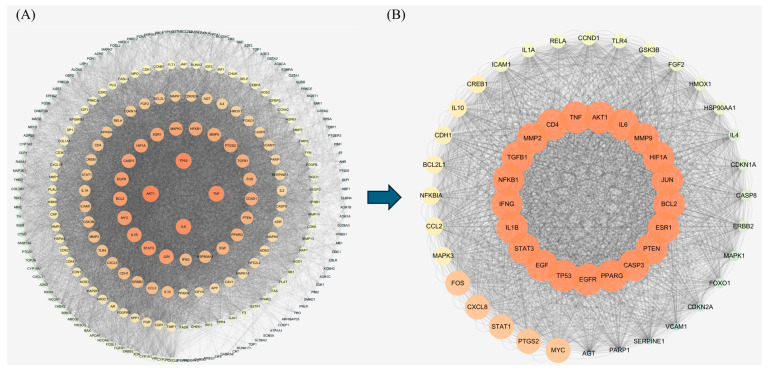
PPI analyses of the Chaga mushroom bioactive compounds before and after screening for potential targets. (**A**) PPI network of all 244 intersection targets of Chaga mushroom bioactive compounds and colorectal cancer. (**B**) PPI network of 53 screened targets based on the median value of BC, CC, and DC. The darker the color, the more important the target. PPI, protein–protein interaction.

String database was used to obtain the protein interaction information between the anti-colorectal cancer targets of Chaga mushroom. These PPI data were imported into Cytoscape 3.7.1 to construct a PPI network of anti-colorectal cancer targets of Chaga mushroom, as shown in Figure 4A, which includes 244 nodes and 8601 interacting edges. In addition, Cytoscape 3.7.1 was also used for the topological analysis of the network, intersecting targets with degree centrality (DC), betweenness centrality (BC), and closeness centrality (CC) values greater than the mean to obtain 53 core targets. These 53 core targets were also subjected to the PPI analysis, as shown in Figure 4B.

**Figure 5:**
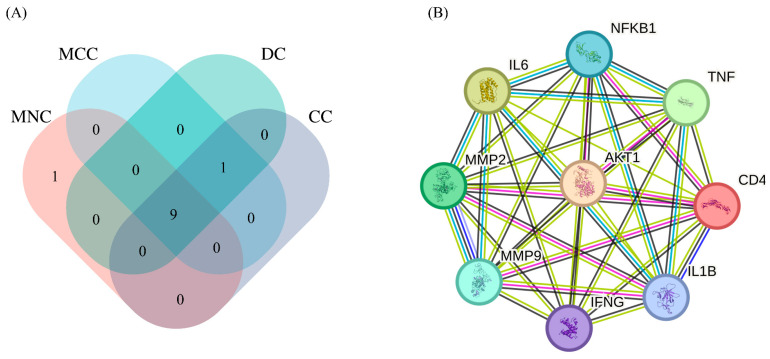
MCC, MNC, DC, and CC screening core targets (**A**) and PPI network of the nine anti-colorectal cancer core targets (**B**). MNC, maximum neighborhood component; MCC, maximal clique centrality; DC, degree centrality; CC, closeness centrality.

To obtain the central target for subsequent molecular docking analysis, we conducted further topological analysis on the core targets and obtained maximum neighborhood component (MNC), maximum clique centrality (MCC), DC, and CC values. According to the intersection analysis of MNC, DC, MCC, and CC values (Figure 5A), the central targets, including AKT1, IFNG, MMP9, MMP2, IL6, NFKB1, TNF, CD4, and IL1B, were identified by intersecting the top 10 targets with different topological values. Moreover, the PPI network of the central target was constructed in Figure 5B. Chaga mushroom can achieve anti-colorectal cancer effects by regulating the central targets.

### 2.4. Results of KEGG and GO Enrichment Analysis

**Figure 6:**
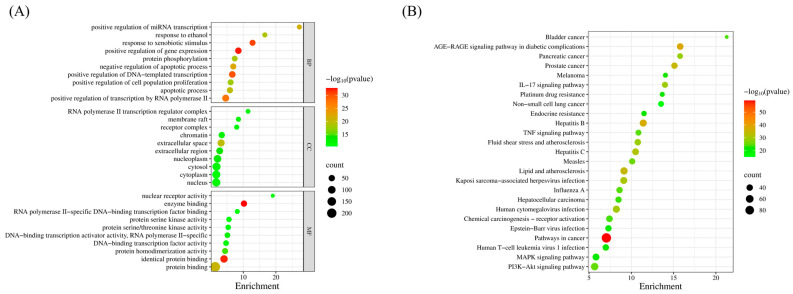
GO (**A**) and KEGG (**B**) enrichment analysis of intersection targets.

The KEGG and GO enrichment analysis was performed based on the anti-colorectal cancer targets of Chaga mushroom in DAVID database (Figure 6). The GO enrichment analysis mainly involved biological processes (BP) including positive regulation of miRNA transcription, response to xenobiotic stimulus, positive regulation of gene expression, protein phosphorylation, negative regulation of apoptotic process, etc. The main cellular components (CC) involved were membrane rafts, receptor complex, chromatin, extracellular space, etc. The molecular function (MF) mainly included nuclear receptor activity, enzyme binding, RNA polymerase II-specific DNA-binding transcription factor binding, etc. Moreover, the KEGG pathway enrichment analysis suggested that the anti-colorectal cancer effect of Chaga mushroom may be achieved through multi-signaling pathway including the AGE-RAGE signaling pathway, IL-17 signaling pathway, TNF signaling pathway, etc.

### 2.5. Results of Molecular Docking

Molecular docking was performed to verify the interaction between the active compounds and the central target in Chaga mushroom. Among them, the active compounds used for docking, the top five compounds with DC values, were selected for molecular docking with the central target. These central targets are AKT1 (4EJN) [22], IFNG (3BES) [23], MMP9 (4XCT) [24], MMP2 (3AYU) [25], IL6 (1ALU) [26], NFKB1 (3GUT) [27], TNF (2AZ5) [22], CD4 (1ALU) [28], and IL1B (1I1B) [28].

**Figure 7:**
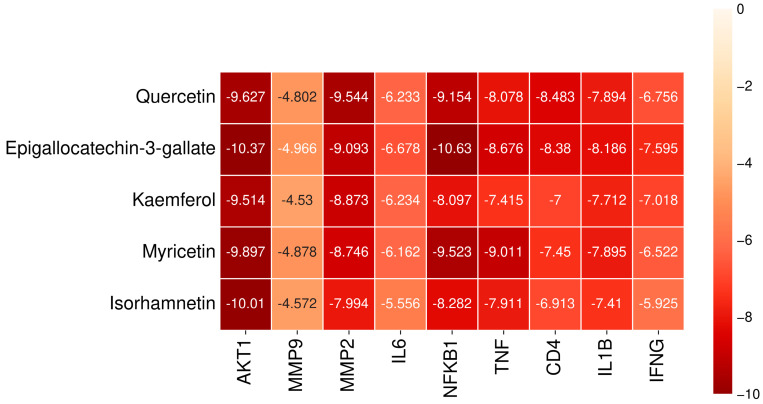
The binding energy network between Chaga mushroom and anti-colorectal cancer targets.

**Figure 8:**
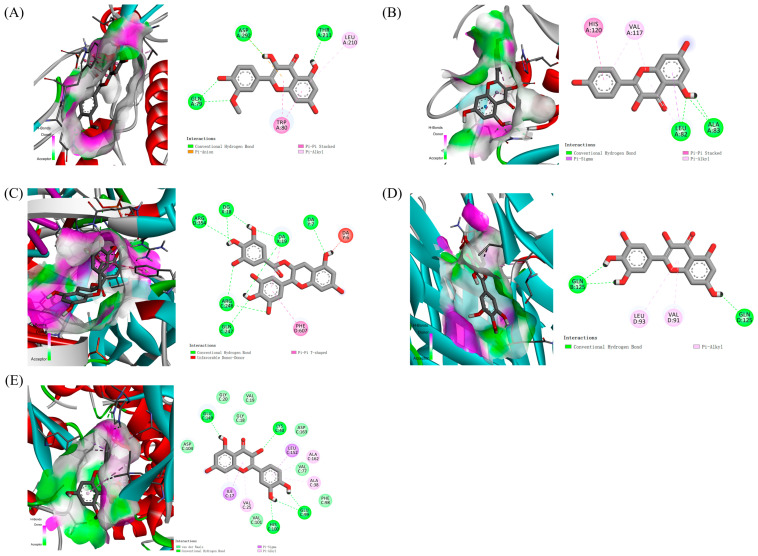
The 2D and 3D diagrams of the docking result between the central targets and compound from Chaga mushroom. (**A**) Isorhamnetin and AKT1. (**B**) Kaempferol and MMP2. (**C**) Epigallocatechin-3-gallate and NFKB1 G complex. (**D**) Myricetin and TNF. (**E**) Quercetin and CD4.

All molecular docking results are shown in Figure 7. Normally, the lower the affinity for protein ligand binding, the more stable the protein ligand docking connection. Specifically, when the binding affinity is less than −5 kcal/mol, the binding affinity between the component and the target is moderate [26]. When the binding affinity is less than −7 kcal/mol, the component target connection is strong [26]. We selected five active compounds with good binding affinity to different targets for visualization analysis (Figure 8). To summarize, the docking between the central target and the active ingredient from Chaga mushroom mainly occurs through multiple interaction forms, including hydrogen bond, van der Waals forces, Pi-Anion, etc.

### 2.6. Results of Bioinformatical Study

To provide a comprehensive understanding of the molecular mechanisms underlying the anticancer effects of Chaga mushroom phytochemicals against colorectal cancer, we conducted an extensive bioinformatics analysis. This analysis included differential gene expression, immune cell infiltration, and survival analysis. The key findings from these analyses are summarized in Table 2, which highlights the integration of these aspects to explain the underlying molecular mechanisms.

#### 2.6.1. Differential Expression Analysis

**Figure 9:**
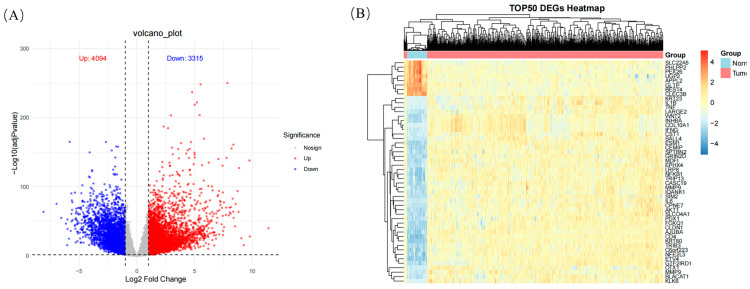
Differential gene expression analysis. (**A**) Differential gene volcano map. (**B**) Top 50 differential gene expressions heatmap.

According to the differential expression analysis results, a total of 7409 differential expression genes (DEGs) were screened, including 3315 significantly upregulated genes and 4094 significantly downregulated genes (Figure 9A). Based on the heat map of differential expression gene analysis, the core genes NFKB1, TNF, CD4, IL1B, IFNG, MMP9, MMP2, IL6, and AKT1 were upregulated in colorectal cancer tumor samples, while SLC22A5, PHLPP2, PEX26, UGP2, APPL2, GLTP, BEST4, and CLEC3B were upregulated in normal tissue samples (Figure 9B).

#### 2.6.2. Immune Infiltration Analysis of Core Gene

**Figure 10:**
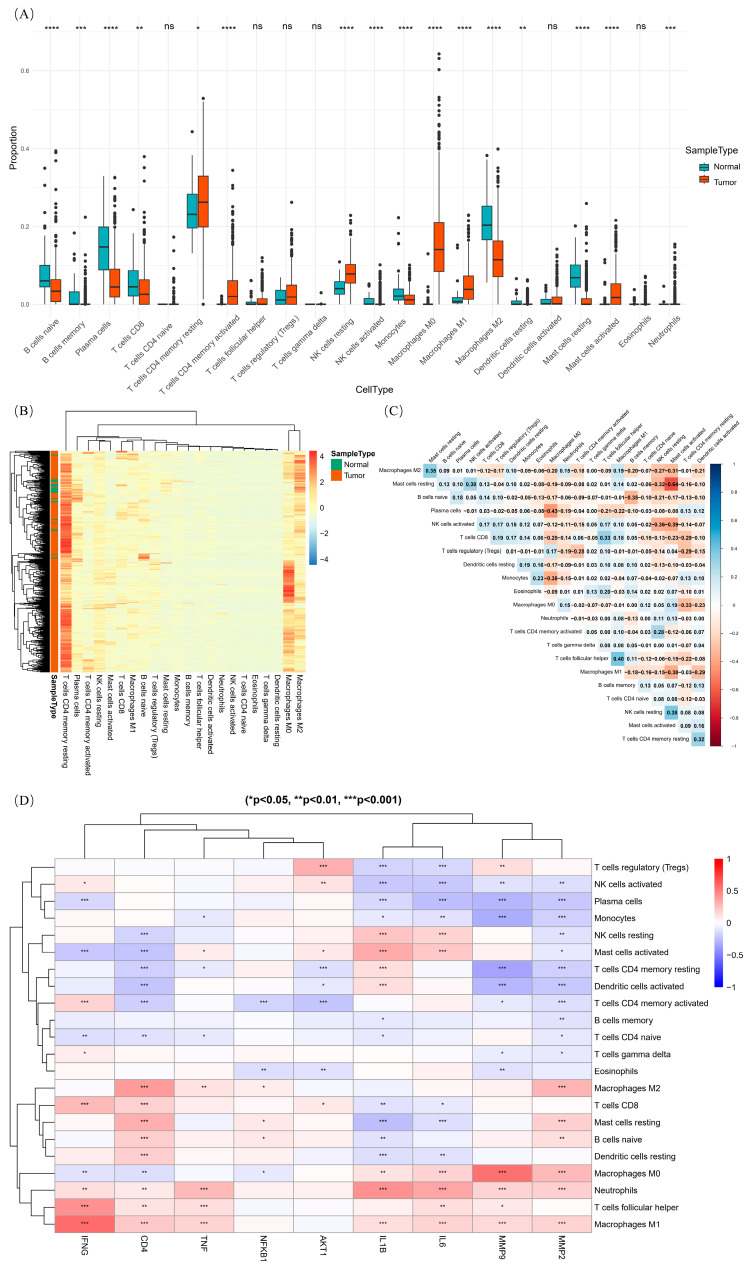
Immune infiltration analysis. (**A**) Immune cell ratio box plot. (**B**) Immune cell infiltration heatmap. (**C**) Correlation diagram of immune infiltration between immune cells. (**D**) Correlation diagram of immune infiltration between core genes and immune cells. “*” means *p*-value < 0.05, “**” means *p*-value < 0.01, “***” means *p*-value < 0.001, and “****” means *p*-value < 0.0001.

The immune cell ratio box plot suggests that there are significant differences in naive B cells, memory B cells, plasma cells, activated CD4 memory T cells, activated NK cells, resting NK cells, etc., between colorectal cancer tumor tissue and normal tissue. In addition, the expressions of resting CD4 memory T cells, resting NK cells, M0 macrophages, M1 macrophages, and hypertrophic resting cells are higher in colorectal cancer tumor tissues than in normal tissues (Figure 10A). According to the immune cell infiltration heatmap, resting CD4 memory T cells, plasma cells, naive B cells, M0 macrophages, and M2 macrophages showed the highest immune infiltration in all samples of TCGA colorectal cancer (Figure 10B). The immune infiltration correlation map between immune cells suggests that the correlation between activated mast cells and resting mast cells is the highest and negatively correlated (−0.64). There is a negative correlation between plasma cells and M0 macrophages, with a degree of −0.43. Furthermore, the correlation between M1 macrophages and follicular immune infiltration is 0.40, indicating a positive correlation (Figure 10C). In colorectal cancer, MMP2, IL1B, IFNG, and CD4 have significant infiltration relationships with many immune cells. Among them, IFNG has the highest immune infiltration correlation with follicular helper T cells and M1 macrophages. The MMP9 gene also has a high immune infiltration correlation with M0 macrophages (Figure 10D).

#### 2.6.3. Survival Analysis of Core Genes

**Figure 11:**
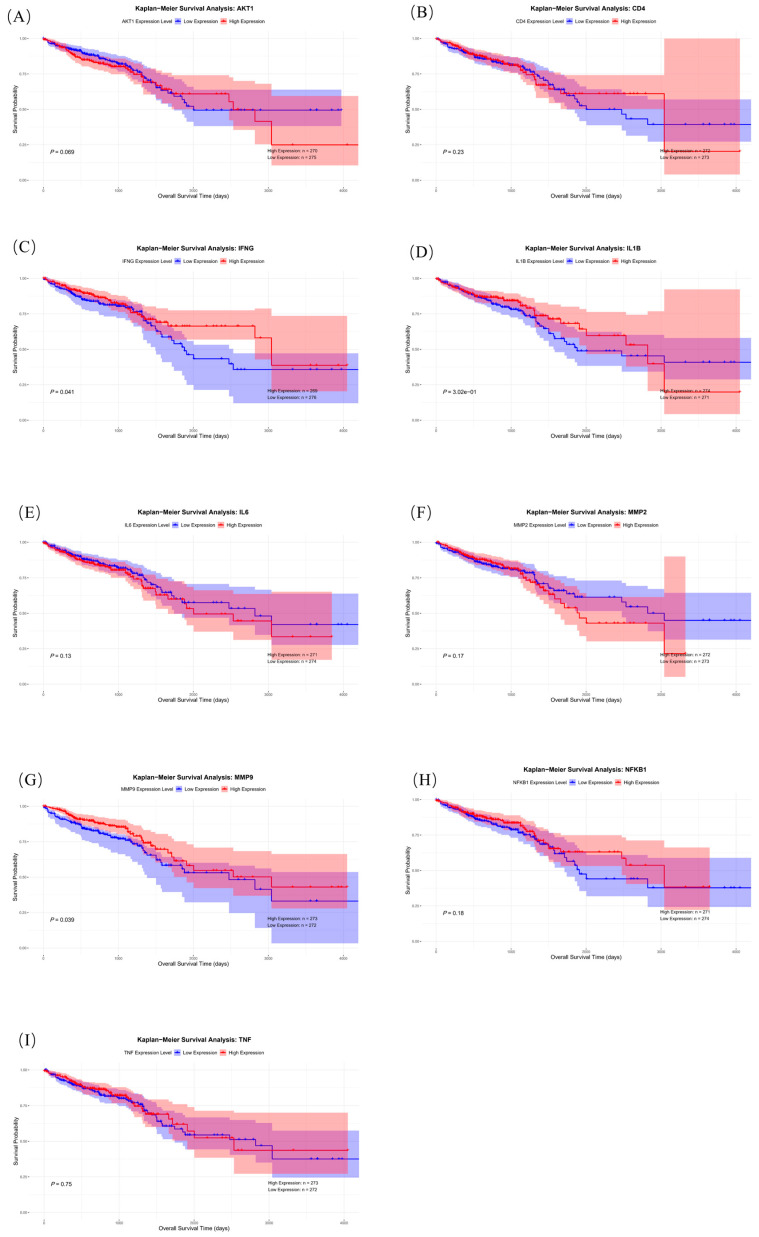
Survival analysis of AKT1 (**A**), CD4 (**B**), IFNG (**C**), IL1B (**D**), IL6 (**E**), MMP2 (**F**), MMP9 (**G**), NFKB1 (**H**), and TNF (**I**).

We conducted survival analysis on core genes to explore the impact of their expression on the prognosis of colorectal cancer patients. The results indicate that the *p*-values of survival analysis for core genes IFNG (Figure 11C), IL1B (Figure 11D), and MMP9 (Figure 11G) are all less than 0.05, suggesting that in colorectal cancer patients, the high expression of IFNG, MMP9, and IL1B indicates a shorter survival time. This indicates that these three core genes have research value in survival prognosis and correspond to the results of immune infiltration analysis.

## 3. Discussion

CRC is the third most common and second-deadliest cancer in the world. So far, CRC has become one of the most important challenges for health systems in many countries. The treatment of this complex disease is mainly based on traditional treatments, including surgery, radiotherapy, and chemotherapy. The high mortality rate indicates that current treatment methods lack success. Therefore, we urgently need to develop efficient and low-toxicity alternative therapies.

Mushrooms have great potential in the field of modern medicine and are favored by many scholars. The use of mushrooms as functional foods and in the treatment of diseases has a long history [29]. Among them, Chaga mushroom (*Inonotus obliquus*) has garnered scientific interest for their potential health benefits. It is reported that Chaga mushroom has characteristics such as immune regulation, antioxidant, anti-inflammatory, and anticancer properties [5]. In recent years, scientists have increasingly focused on the anticancer potential and bioactivity of Chaga mushroom. However, most studies have focused on its antioxidant and anti-inflammatory properties, with limited research on its specific mechanisms in CRC treatment. Our study fills this gap by providing a comprehensive analysis of the molecular mechanisms of Chaga mushroom phytochemicals against CRC.

In our study, by searching different databases for the active ingredients in Chaga mushroom, 26 active ingredients were finally obtained. Similarly, 5064 colorectal cancer-related targets were obtained, resulting in 244 potential therapeutic targets. After PPI analysis and further screening, we collected nine core targets, including NFKB1, TNF, CD4, IL1B, IFNG, MMP9, MMP2, IL6, and AKT1. In addition, the GO enrichment analysis mainly involved biological processes (BPs), including positive regulation of miRNA transcription, response to xenobiotic stimulus, positive regulation of gene expression, protein phosphorylation, negative regulation of the apoptotic process, etc. The main cellular components (CC) involved were membrane raft, receptor complex, chromatin, extracellular space, etc. The molecular function (MF) mainly included nuclear receptor activity, enzyme binding, RNA polymerase II-specific DNA-binding transcription factor binding, etc. Moreover, the KEGG pathway enrichment analysis suggested that the anti-colorectal cancer effect of Chaga mushroom may be achieved through multi-signaling pathway including the AGE-RAGE signaling pathway, IL-17 signaling pathway, TNF signaling pathway, etc. Furthermore, we also conducted the molecular docking of core targets and top five bioactive compounds (quercetin, epigallocatechin-3-gallate, kaempferol, myricetin, and isorhamnetin) and found that the docking between the central target and the active ingredient from Chaga mushroom mainly occurs through multiple interaction forms, including hydrogen bond, van der Waals forces, Pi-Anion, etc. Subsequently, we explored the impact of core targets on colorectal cancer using bioinformatics studies. Our results indicate that there are significant differences in naive B cells, memory B cells, plasma cells, activated CD4 memory T cells, activated NK cells, resting NK cells, etc., between colorectal cancer tumor tissue and normal tissue. In colorectal cancer, MMP2, IL1B, IFNG, and CD4 have significant infiltration relationships with many immune cells. Among them, IFNG has the highest immune infiltration correlation with follicular helper T cells and M1 macrophages. The MMP9 gene also has a high immune infiltration correlation with M0 macrophages. Survival analysis of core genes suggests that high expression of IFNG, MMP9, and IL1B indicates a shorter survival time in colorectal cancer patients.

According to previous research, when the binding affinity was less than −5 kcal/mol, the binding between component and target was moderate. The component–target connection was strong when the binding affinity was less than −7 kcal/mol [26]. Our research suggests that the binding affinities between most of the bioactive compounds of Chaga mushrooms and the core proteins are good, with binding energies less than −5 kcal/mol. Among them, the bioactive compounds of Chaga mushrooms have a strong binding effect with AKT1, with binding energies less than −7 kcal/mol. Overexpression of the AKT-activated PI3K/AKT signaling pathway is a common molecular attribute of several cancers. Hence, targeting AKT appears to be a potential therapeutic option, and direct inhibition of AKT kinase activity can potentially attenuate cancer growth [30]. A study computationally investigated a few phytochemical flavonoids to establish their mechanism of action and potential to inhibit AKT1 [31] and showed that tehranolide has a greater capacity to inhibit AKT1 expression, which may be useful in the creation of anticancer drugs because AKT1 inhibition is directly linked to the prevention of tumor growth, progression, and metastasis. Comparing the binding affinity of tehranolide and AT1 (binding energy is −9.22 kcal/mol), we found that the top five active compounds of Chaga mushrooms in our study show lower binding energy with the range from −9.514 to −10.37 kcal/mol through multiple interaction forms, including hydrogen bond, van der Waals forces, Pi-Anion, etc.

Polyphenols, such as quercetin, play multiple positive roles in maintaining cellular oxidative and inflammatory abilities, which makes them potentially useful as anticancer drugs. There is a clear correlation between quercetin and the interference of Wnt, P13K/AKT, caspase-3, MAPK, NF-κ B and other mechanisms pathways involved in CRC [32]. The anti-tumor properties of epigallocatechin gallate (EGCG), a naturally occurring polyphenol that is isolated from tea leaves, have drawn a lot of interest. By inducing cell death, preventing invasion, migration, and proliferation, changing the epigenetic alterations of tumors, and defeating chemotherapy resistance, EGCG has been shown in an increasing number of studies to prevent the development and spread of tumors. Additionally, EGCG can improve the effectiveness of immunotherapy and could be a good option for immunotherapy against tumors [33]. Kaempferol is a naturally occurring flavonoid component that has been discovered in a variety of fruits and vegetables in recent years. It has drawn a lot of interest in gastrointestinal cancer research because of its various therapeutic benefits. Inhibiting cell proliferation, survival, angiogenesis, metastasis, and migration, kaempferol has been demonstrated to modify several molecular mechanisms and pathways, including the PI3/Akt, mTOR, and Erk/MAPK pathways implicated in the advancement of cancer [34]. Myricetin has been found to effectively inhibit the proliferation of different colon cancer cell lines. The mechanism may be related to inhibiting the PI3K/Akt/mTOR signaling pathway to induce cell apoptosis and autophagy [35]. The chemical protective effect of isorhamnetin on colon cancer is related to its anti-inflammatory activity, inhibition of carcinogenic Src activity, and corresponding loss of nuclear β-catenin, which depend on the expression of C-terminal Src kinase (CSK) [36].

According to survival analysis of core genes, colorectal cancer patients with high levels of IFNG, MMP9, and IL1B expression are likely to have shorter survival times. According to a study examining the immune gene expression profiles of several CRC patient subgroups, the most immunogenic subgroup of CRC patients had higher levels of KLRK1, which was positively connected with IFNG mRNA expression and linked to a worse chance of survival [37]. The tumor microenvironment’s (TME) dynamic alterations are what propel colorectal cancer’s development and spread. The extracellular matrix (ECM) is a major player in complicated TME, where alterations in composition, hardness, and disintegration are thought to be important determinants in tumor growth. Reports suggest that dyscadherin/matrix metalloprotease 9 (MMP9) axis may be a viable therapeutic target for colorectal cancer (CRC) since it can accelerate the disease’s progression by altering TME through ECM remodeling [38]. In cancer cells and aggressive tumors, IL-1B usually stimulates angiogenesis, immune cell proliferation, and invasion. IL-1B was found to be significantly elevated in the mice model of adenomatous polyposis coli (APC) cancer. Recent research has demonstrated that while the lack of IL-1R1-in epithelial cells lowers tumor incidence in APC models, the absence of IL-1R1-in neutrophils increases bacterial invasion and tumor invasiveness [28].

Although this study comprehensively explored the molecular mechanisms of Chaga mushroom against colorectal cancer through methods such as network pharmacology, molecular docking, and bioinformatics, there are still some limitations. The results of network pharmacology and bioinformatics analysis need to be validated through in vitro and in vivo experiments if condition is permitted. Molecular docking only involves some compounds targeting the target, which may overlook other potential interactions. Our study also lacks an in-depth comparison of anti-tumor mechanisms in different cancers. In the future, experiments are needed to verify and explore the roles in various types of cancer, providing a more comprehensive basis for treatment. For example, cytotoxicity assays on CRC cell lines (e.g., HT-29, SW480) should be performed to determine the IC50 values of selected Chaga mushroom phytochemicals; cell proliferation assays (e.g., MTT or CCK-8 assays) should be conducted to investigate the effects of these phytochemicals on CRC cell growth; AKT1 inhibition assays can be performed to evaluate the ability of these compounds to inhibit AKT1 activity, a key protein involved in cancer progression. Following successful in vitro validation, in vivo studies using appropriate animal models can be used to assess the therapeutic efficacy and safety of phytochemicals derived from Chaga mushroom in a more biologically relevant context. We believe that these experimental validations will significantly enhance the translational impact of our study.

## 4. Materials and Methods

### 4.1. Collection of Active Components and Targets of Chaga Mushroom (Inonotus obliquus)

All chemical components from the Chaga mushroom were collected from the literature [39]. The structures of these compounds were retrieved from the PubChem database (https://pubchem.ncbi.nlm.nih.gov/, accessed on 2 January 2025). According to the five principles of drug similarity (relative molecular weight < 500, number of hydrogen bond donors < 5, number of hydrogen bond acceptors < 10, lipid water partition coefficient < 5, number of rotatable bonds ≤ 10), compounds that met three or more of these principles were considered as active compounds [40], and potential target proteins were predicted using the SWISStargetprediction database (http://swisstargetprediction.ch/, accessed on 2 January 2025). Then, the obtained target proteins were standardized using the Uniprot protein database (https://www.uniprot.org/, accessed on 2 January 2025) [41].

### 4.2. Collection of Colorectal Cancer Targets

The disease targets were collected using the keywords “colorectal cancer” and “colon cancer” in the Genecard database (https://www.genecards.org/, accessed on 5 January 2025), OMIM database (https://www.omim.org/, accessed on 5 January 2025), and DurgBank database. Moreover, the obtained targets underwent de-duplication processing and standardization of protein information through the UniProt database (accessed on 5 January 2025) [28].

### 4.3. Collection of Targets for the Treatment of Colorectal Cancer by Chaga Mushroom

The Venne diagram was used to obtain targets for the treatment of colorectal cancer by Chaga mushroom. The intersection analysis between the targets of the active ingredient derived from Chaga mushroom and the colorectal cancer targets yields the intersection targets that are related to the treatment of colorectal cancer by Chaga mushroom. In addition, the Cytoscape 3.7.1 software was used to construct a drug-compound-target-disease network to describe the mechanism of Chaga mushroom in treating colorectal cancer [28].

### 4.4. Protein–Protein Interaction (PPI) Analysis of the Anti-Colorectal Cancer Targets of Chaga Mushroom

The anti-colorectal cancer targets of Chaga mushroom were imported into the STRING database (https://cn.string-db.org/, accessed on 10 January 2025) for the protein–protein interaction analysis between each target. The species is designed to be set as ‘Homo sapiens’, with a confidence level set to higher than 0.4. Moreover, in order to construct a visual PPI network, the data were imported into Cytoscape 3.7.2 for network construction [41].

### 4.5. The Screening of the Core Targets and Central Targets

BisoGenet and CytoNCA plugins were utilized for analyzing drug targets and disease targets. The targets with DC, BC, and CC greater than the mean were taken to interact and identify as the core target. Moreover, the targets with top 10 MNC, MCC, DC, and CC value were calculated by the cytohubba software in Cytoscape 3.7.2 and taken to interact and identify the interaction targets as the central targets [22].

### 4.6. The KEGG and GO Analysis

The DAVID database (https://davidbioinformatics.nih.gov/, accessed on 10 January 2025) was used for GO and KEGG enrichment analysis of the core anti-colorectal cancer targets of Chaga mushroom. The identifier and species were selected as “OFFICIL-GENE-SYMBOL” and “HOMO Sapiens”. For the KEGG and GO enrichment analysis, the top 25 pathways of the targets were selected based on *p*-values, and the results were visualized using R software (Version 4.4.1) [22].

### 4.7. Molecular Docking

The structures of bioactive compounds in Chaga mushroom were imported into Chem3D (Version 22.0) to optimize their 3D structure using the MM2 energy-minimized model, which was then used as ligands in molecular docking. The central target was selected as the receptor for molecular docking. The protein receptors were selected based on the literature, and the resolution downloaded from the PDB database (https://www.rcsb.org/, accessed on 12 January 2025) [42]. The protein targets were imported into the AutoDockTools (Version 1.5.7) for pre-processing, including removing water and adding hydrogen. The molecular docking was conducted by the AutoDock Vina (Version 1.2.3) and the grid box covered the whole protein receptor. The detailed information about molecular docking is recorded in Table S1.

### 4.8. Bioinformatical Study

We download gene expression data and clinical information for colon cancer (COAD) and rectal cancer (READ) from the Cancer Genome Atlas Project (TCGA) (https://portal.gdc.cancer.gov/, accessed on 4 February 2025), and integrate these cancer data for subsequent analysis [43].

All transcriptome data were standardized and normalized and analyzed using the “limma” software package (version 3.5.1, accessed on 6 February 2025). The DEGs threshold was set as follows: log2FoldChange > 1, adjusted *p*-value < 0.05 [44]. Volcano and heatmaps were created using the “ggplot2” and “pheatmap” packages for visualizing DEGs [45]. In addition, this study used the CIBERSORT algorithm to quantitatively analyze the immune infiltration of tumor samples [45]. Subsequently, this study conducted survival analysis based on the expression levels of target genes to evaluate their potential impact on patient prognosis. Used the survfit() function to fit the survival curve, and calculate the *p*-value of the log rank test using the survdiff() function to test the statistical significance of survival differences between different expression groups. In all analyses, if the *p*-value was less than 0.05, the difference was considered statistically significant [46].

## 5. Conclusions

In summary, this study used network pharmacology, molecular docking, and bioinformatics to elucidate the molecular mechanisms of phytochemicals from Chaga mushroom (*Inonotus obliquus*) against colorectal cancer. Our findings identified several bioactive compounds and their potential targets, highlighting key pathways and signaling networks involved in the anti-colorectal cancer effects. The core targets, including AKT1, IFNG, MMP9, MMP2, IL6, NFKB1, TNF, CD4, and IL1B, were found to be crucial in mediating the anticancer activities of Chaga mushroom. Molecular docking studies confirmed the binding affinities of these compounds to their respective targets, further supporting their potential therapeutic roles. Additionally, bioinformatics analysis revealed significant differential gene expression and immune infiltration patterns associated with these targets, suggesting their importance in the tumor microenvironment and patient prognosis. Despite the limitations of computational predictions requiring experimental validation, our study provides a comprehensive framework for understanding the multifaceted actions of Chaga mushroom phytochemicals and offers valuable findings for the development of novel therapeutic strategies against colorectal cancer. Future research should focus on experimental validation of these findings and exploration of the broader anticancer potential of Chaga mushroom in diverse cancer contexts.

## Figures and Tables

**Table 1:** Bioactive compounds of Chaga mushroom.

PubChem Name	CID Number	Molecular Formula	Structure	References
Quercetin	5280343	C_15_H_10_O_7_	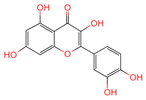	[13]
Epigallocatechin-3-gallate	65064	C_22_H_18_O_11_	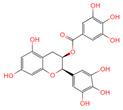	[14]
Kaempferol	5280863	C_15_H_10_O_6_	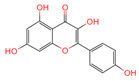	[15]
Myricetin	5281672	C_15_H_10_O_8_	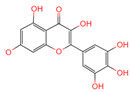	[16]
Isorhamnetin	5281654	C_16_H_12_O_7_	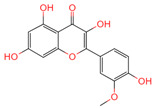	[16]
Salicylic acid	338	C_7_H_6_O_3_	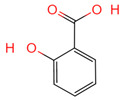	[16]
Tangeretin	68077	C_20_H_20_O_7_	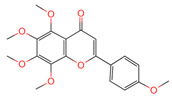	[15]
Betulinic acid	64971	C_30_H_48_O_3_	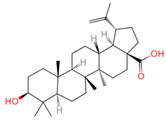	[15]
Oleanolic acid	10494	C_30_H_48_O_3_	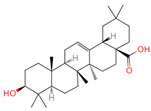	[17]
Ellagic acid	5281855	C_14_H_6_O_8_	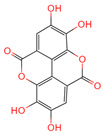	[16]
3,4,5-Trihydroxybenzoic acid	370	C_7_H_26_O_5_	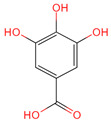	[18]
Protocatechuic aldehyde	8768	C_7_H_6_O_3_	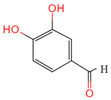	[19]
Ferulic acid	445858	C_10_H_10_O_4_	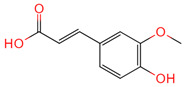	[14]
Catechin	9064	C_15_H_14_O_6_	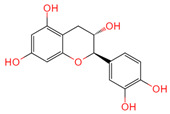	[19]
Protocatechuic acid	72	C_7_H_6_O_4_	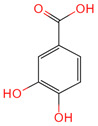	[16]
p-Coumaric acid	637542	C_9_H_8_O_3_	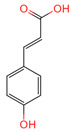	[15]
Ergosterol	444679	C_28_H_44_O	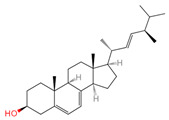	[20]
Vanillic acid	8468	C_8_H_8_O_4_	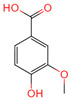	[16]
Caffeate	689043	C_9_H_8_O_4_	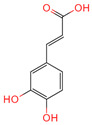	[15]
p-MCA	699414	C_10_H_10_O_3_	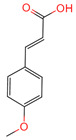	[16]
Lanosterol	246983	C_30_H_50_O	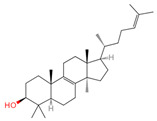	[20]
Ergosterol peroxide	5351516	C_28_H_44_O_3_	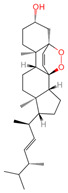	[21]
Trametenolic acid	12309443	C_30_H_48_O_3_	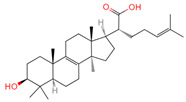	[15]
Epicatechin gallate	107905	C_22_H_18_O_10_	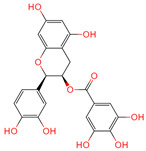	[14]
Cedar acid	10742	C_9_H_10_O_5_	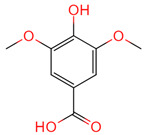	[18]

**Table 2:** Summary of bioinformatics analysis findings.

Aspect	Key Findings	Implications
Differential gene expression	7409 DEGs identified, including 3315 upregulated and 4094 downregulated genes	Core genes (e.g., IFNG, IL1B, MMP9) are upregulated in tumor samples, indicating their role in cancer progression.
Immune cell infiltration	Significant differences in immune cell infiltration between tumor and normal tissues	Immune cells such as CD4+ T cells, M0 macrophages, and M2 macrophages show higher infiltration in tumor tissues, correlating with core genes like IFNG and MMP9.
Survival analysis	High expression of IFNG, MMP9, and IL1B indicates shorter survival time in colorectal cancer patients	These core genes are potential biomarkers for prognosis and therapeutic targets.

## Data Availability

The data presented in this study are available on request from the corresponding author.

## Supplementary Materials

The following supporting information can be downloaded at https://www.mdpi.com/article/10.3390/ijms26167664/s1.

## Abbreviations

BC	Betweenness centrality
BP	Biological process
CC	Cellular component
CRC	Colorectal cancer
DC	Degree centrality
DEGs	Differential expression genes
DL	Drug-likeness
ECM	Extracellular matrix
GO	Gene Ontology
IL1B	Interleukin lB
KEGG	Kyoto Encyclopedia of Genes and Genomes
MF	Molecular function
MCC	Maximum Clique Centrality
MMP	Matrix metalloprotease 9
MNC	Maximum Neighborhood Component
PDB	Protein Data Bank
PPI	Protein–protein Interaction
TME	Tumor microenvironment
