# Supplementary material for: Molecular Mechanisms of Phytochemicals from Chaga Mushroom (Inonotus obliquus) Against Colorectal Cancer: Insights from Network Pharmacology, Molecular Docking, and Bioinformatics
Source: Int J Mol Sci. 2025 Aug 8;26(16):7664. doi: 10.3390/ijms26167664 (PMC12386236; doi:10.3390/ijms26167664)
Supplement: Supplementary file 1 [file ijms-26-07664-s001.zip › ijms-3767972-supplementary.pdf]

Table S1. Detailed information about molecular docking.

No.	Ligand	Protein	Grid Center X	Grid Center Y	Grid Center Z	Grid size X	Grid size Y	Grid size Z	Grid space	Affinity (kcal/mol)
1	Quercetin	4ejn_AKT1	32.053	41.234	13.57	60.0	76.0	94.0	0.375	-9.627
2	Epigallocatechin-3-gallate	4ejn_AKT1	32.053	41.234	13.57	60.0	76.0	94.0	0.375	-10.37
3	Kaemferol	4ejn_AKT1	32.053	41.234	13.57	60.0	76.0	94.0	0.375	-9.514
4	Myricetin	4ejn_AKT1	32.053	41.234	13.57	60.0	76.0	94.0	0.375	-9.897
5	Isorhamnetin	4ejn_AKT1	32.053	41.234	13.57	60.0	76.0	94.0	0.375	-10.01
6	Quercetin	4xct_MMP9	0.0	0.0	0.0	15.0	15.0	15.0	0.375	-4.802
7	Epigallocatechin-3-gallate	4xct_MMP9	0.0	0.0	0.0	15.0	15.0	15.0	0.375	-4.966
8	Kaemferol	4xct_MMP9	0.0	0.0	0.0	15.0	15.0	15.0	0.375	-4.53
9	Myricetin	4xct_MMP9	0.0	0.0	0.0	15.0	15.0	15.0	0.375	-4.878
10	Isorhamnetin	4xct_MMP9	0.0	0.0	0.0	15.0	15.0	15.0	0.375	-4.572
11	Quercetin	3ayu_MMP2	1.375	-11.845	-4.572	78.0	64.0	58.0	0.375	-9.544
12	Epigallocatechin-3-gallate	3ayu_MMP2	1.375	-11.845	-4.572	78.0	64.0	58.0	0.375	-9.093
13	Kaemferol	3ayu_MMP2	1.375	-11.845	-4.572	78.0	64.0	58.0	0.375	-8.873
14	Myricetin	3ayu_MMP2	1.375	-11.845	-4.572	78.0	64.0	58.0	0.375	-8.746
15	Isorhamnetin	3ayu_MMP2	1.375	-11.845	-4.572	78.0	64.0	58.0	0.375	-7.994
16	Quercetin	1alu_IL6	0.0	0.0	0.0	64.0	68.0	58.0	0.375	-6.233
17	Epigallocatechin-3-gallate	1alu_IL6	0.0	0.0	0.0	64.0	68.0	58.0	0.375	-6.678
18	Kaemferol	1alu_IL6	0.0	0.0	0.0	64.0	68.0	58.0	0.375	-6.234
19	Myricetin	1alu_IL6	0.0	0.0	0.0	64.0	68.0	58.0	0.375	-6.162
20	Isorhamnetin	1alu_IL6	0.0	0.0	0.0	64.0	68.0	58.0	0.375	-5.556
21	Quercetin	3gut_NFKB1	41.883	42.22	43.361	126.0	126.0	126.0	0.375	-9.154
22	Epigallocatechin-3-gallate	3gut_NFKB1	41.883	42.22	43.361	126.0	126.0	126.0	0.375	-10.63
23	Kaemferol	3gut_NFKB1	41.883	42.22	43.361	126.0	126.0	126.0	0.375	-8.097
24	Myricetin	3gut_NFKB1	41.883	42.22	43.361	126.0	126.0	126.0	0.375	-9.523
25	Isorhamnetin	3gut_NFKB1	41.883	42.22	43.361	126.0	126.0	126.0	0.375	-8.282
26	Quercetin	2az5_TNF	-13.687	71.608	27.0	88.0	86.0	104.0	0.375	-8.078
27	Epigallocatechin-3-gallate	2az5_TNF	-13.687	71.608	27.0	88.0	86.0	104.0	0.375	-8.676
28	Kaemferol	2az5_TNF	-13.687	71.608	27.0	88.0	86.0	104.0	0.375	-7.415
29	Myricetin	2az5_TNF	-13.687	71.608	27.0	88.0	86.0	104.0	0.375	-9.011
30	Isorhamnetin	2az5_TNF	-13.687	71.608	27.0	88.0	86.0	104.0	0.375	-7.911
31	Quercetin	3g33_CD4	-5.583	-32.701	-50.12	126.0	126.0	108.0	0.375	-8.483
32	Epigallocatechin-3-gallate	3g33_CD4	-5.583	-32.701	-50.12	126.0	126.0	108.0	0.375	-8.38
33	Kaemferol	3g33_CD4	-5.583	-32.701	-50.12	126.0	126.0	108.0	0.375	-7
34	Myricetin	3g33_CD4	-5.583	-32.701	-50.12	126.0	126.0	108.0	0.375	-7.45
35	Isorhamnetin	3g33_CD4	-5.583	-32.701	-50.12	126.0	126.0	108.0	0.375	-6.913
36	Quercetin	1i1b_IL1B	41.423	-15.648	23.361	70.0	58.0	58.0	0.375	-7.894
37	Epigallocatechin-3-gallate	1i1b_IL1B	41.423	-15.648	23.361	70.0	58.0	58.0	0.375	-8.186

38	Kaemferol	1i1b_IL1B	41.423	-15.648	23.361	70.0	58.0	58.0	0.375	-7.712
39	Myricetin	1i1b_IL1B	41.423	-15.648	23.361	70.0	58.0	58.0	0.375	-7.895
40	Isorhamnetin	1i1b_IL1B	41.423	-15.648	23.361	70.0	58.0	58.0	0.375	-7.41
41	Quercetin	3bes_IFNG	38.96	41.993	30.333	121.45	119.522	117.594	0.375	-6.756
42	Epigallocatechin-3-gallate	3bes_IFNG	38.96	41.993	30.333	121.45	119.522	117.594	0.375	-7.595
43	Kaemferol	3bes_IFNG	38.96	41.993	30.333	121.45	119.522	117.594	0.375	-7.018
44	Myricetin	3bes_IFNG	38.96	41.993	30.333	121.45	119.522	117.594	0.375	-6.522
45	Isorhamnetin	3bes_IFNG	38.96	41.993	30.333	121.45	119.522	117.594	0.375	-5.925
